# Thickness dependence of the triplet spin-valve effect in superconductor–ferromagnet–ferromagnet heterostructures

**DOI:** 10.3762/bjnano.7.88

**Published:** 2016-07-04

**Authors:** Daniel Lenk, Vladimir I Zdravkov, Jan-Michael Kehrle, Günter Obermeier, Aladin Ullrich, Roman Morari, Hans-Albrecht Krug von Nidda, Claus Müller, Mikhail Yu Kupriyanov, Anatolie S Sidorenko, Siegfried Horn, Rafael G Deminov, Lenar R Tagirov, Reinhard Tidecks

**Affiliations:** 1 Institut für Physik, Universität Augsburg, Universitätsstraße 1, D-86159 Augsburg, Germany; 2 D. Ghitsu Institute of Electronic Engineering and Nanotechnologies ASM, Academiei Str. 3/3, MD2028 Kishinev, Moldova; 3 Solid State Physics Department, Kazan Federal University, Kremlevskaya Str. 18, 420008 Kazan, Russian Federation; 4 Skobeltsyn Institute of Nuclear Physics, Moscow State University, Leninskie gory, GSP-1, Moscow 119992, Russia

**Keywords:** heterostructures, superconducting spin valve, thin films, triplet superconductivity

## Abstract

**Background:** In nanoscale layered S/F_1_/N/F_2_/AF heterostructures, the generation of a long-range, odd-in-frequency spin-projection one triplet component of superconductivity, arising at non-collinear alignment of the magnetizations of F_1_ and F_2_, exhausts the singlet state. This yields the possibility of a global minimum of the superconducting transition temperature *T*_c_, i.e., a superconducting triplet spin-valve effect, around mutually perpendicular alignment.

**Results:** The superconducting triplet spin valve is realized with S = Nb a singlet superconductor, F_1_ = Cu_41_Ni_59_ and F_2_ = Co ferromagnetic metals, AF = CoO*_x_* an antiferromagnetic oxide, and N = nc-Nb a normal conducting (nc) non-magnetic metal, which serves to decouple F_1_ and F_2_. The non-collinear alignment of the magnetizations is obtained by applying an external magnetic field parallel to the layers of the heterostructure and exploiting the intrinsic perpendicular easy-axis of the magnetization of the Cu_41_Ni_59_ thin film in conjunction with the exchange bias between CoO*_x_* and Co. The magnetic configurations are confirmed by superconducting quantum interference device (SQUID) magnetic moment measurements. The triplet spin-valve effect has been investigated for different layer thicknesses, *d*_F1_, of F_1_ and was found to decay with increasing *d*_F1_. The data is described by an empirical model and, moreover, by calculations using the microscopic theory.

**Conclusion:** The long-range triplet component of superconducting pairing is generated from the singlet component mainly at the N/F_2_ interface, where the amplitude of the singlet component is suppressed exponentially with increasing distance *d*_F1_. The decay length of the empirical model is found to be comparable to twice the electron mean free path of F_1_ and, thus, to the decay length of the singlet component in F_1_. Moreover, the obtained data is in qualitative agreement with the microscopic theory, which, however, predicts a (not investigated) breakdown of the triplet spin-valve effect for *d*_F1_ smaller than 0.3 to 0.4 times the magnetic coherence length, ξ_F1_.

## Introduction

Fulde and Ferrell [1], and Larkin and Ovchinnikov [2] (FFLO) predicted superconductivity on a ferromagnetic background, i.e., in the presence of an exchange field. This was unexpected, because singlet superconductivity is established by pairs of electrons (Cooper pairs) with anti-parallel spin [3], but ferromagnetism leads to a parallel alignment of the electron spins. Indeed, experimental realizations of the FFLO state are scarce [4]. However, a quasi-one-dimensional FFLO-like state can be realized in thin-film superconductor (S)/ferromagnet (F) proximity-effect systems [5–7].

Here, singlet Cooper pairs in the F-material are formed with zero total spin but non-zero total momentum. This leads to a pairing wave function (PWF), which decays exponentially and oscillates as a function of space [5–7]. As a consequence, interference effects occur, if the PWF is reflected at the outer border of the F-layer of a S/F bilayer, yielding an oscillating superconducting transition temperature as a function of the F-layer thickness, even an extinction of superconductivity with a subsequent recovery (reentrant behavior) is observed [7–9].

Because for two F-layers the superconducting transition temperature depends on the relative orientation of their magnetizations [9–16], trilayers of F/S/F-type and S/F/F-type represent superconducting spin-valves (SSVs) [10–12,15–16]. While the SSV effect in F/S/F structures has been extensively investigated [17–35], this has only been done to a lesser degree in S/F/F-type structures [36–40]. However, to realize the triplet SSV effect, i.e., an absolute minimum of the superconducting transition temperature, *T*_c_, at non-collinear magnetizations [15], the S/F/F-type SSV is most suitable, due to the spatial vicinity of the two F-layers [41–47]. Although the dirty-limit theory for the F/S/F-type SSV predicts a contribution of the spin-projection one triplet component to the superconducting *T*_c_, a triplet SSV effect is absent [14,16]. However, the triplet SSV effect was experimentally realized in F/S/F-type SSVs using elemental ferromagnets [46,48] and has been recently predicted by theory for clean ferromagnets [16].

Without triplet pairing states, the dependence of *T*_c_ on the relative magnetic orientation of the F-layers, e.g., in an F/S/F heterostructure, would not be present (at least in the dirty limit). The reason is that the singlet component does not carry information about the direction of the magnetization, which it is suppressed by [19]. Therefore, the triplet components play a crucial role in S/F proximity-effect devices. In the F-material, pairing states of electrons with opposite spins can be superimposed antisymmetrically to a s-wave singlet and symmetrically to a s-wave triplet spin state with zero spin projection, respectively [49]. Equal-spin pairing yields s-wave triplet pairing states with spin-projection one, which are odd in Matsubara frequency [6,14–15,49–51] like the triplet state with zero spin projection [49,51]. Because of the equal spin pairing, the antagonism between the spin ordering of the ferromagnetism and superconductivity is lifted and, thus, the corresponding states are of extraordinarily long range in space and robust against scattering at non-magnetic impurities [49,51] (for the discussion of the decay length see also [52]).

However, only the singlet component enters the self-consistency equation for the superconducting gap and is, thus, the only component directly affecting *T*_c_ [14–15]. For uniform magnetization of the F-layer in a S/F bilayer and collinear magnetizations in F/S/F and S/F/F trilayers, the singlet and the triplet component with spin-projection zero exist and are coupled with each other [14–15,49]. For non-collinear magnetizations, the long-range spin-projection one triplet component is generated, which is coupled to the zero spin-projection triplet and singlet component and, thus, indirectly enters the self-consistency equation [14–15], yielding a change of the superconducting gap and *T*_c_. A descriptive picture of the triplet SSV effect is that the generation of the triplet Cooper pairs exhausts the singlet state and, thus, suppresses *T*_c_. This yields the possibility of an absolute minimum of the transition temperature as a function of the angle between the magnetizations of F_1_ and F_2_ near the perpendicular orientation [15].

To achieve a non-collinear alignment of the magnetizations of the two F-layers in our S/F_1_/F_2_-type SSV, we exploit the unusual perpendicular easy-axis of magnetization of thin Cu_41_Ni_59_ films [41,53–54] (used as F_1_ layer material). After cooling the samples in a magnetic field applied parallel to the film plane, the magnetization of the F_2_ layer (a thin Co film) is pinned in the cooling-field direction (in-plane) by the exchange bias interaction with a CoO*_x_* film. This shifts the coercive field of the Co film, *H*_c,Co_, to high negative fields with respect to the cooling-field direction, yielding clear separation of the coercive fields of the two ferromagnetic layers [55]. At the small negative coercive field of the Cu_41_Ni_59_ film, *H*_c,CuNi_, the Co film is still in the mono-domain state with in-plane magnetization, while the Cu_41_Ni_59_ film is expected to exhibit a local net magnetization along its easy axis perpendicular to the layer (although the total magnetic moment of the layer is zero).

Thus, with a magnetic field applied parallel to the film plane, we are able to change the magnetic configuration of our Nb/Cu_41_Ni_59_/nc-Nb/Co/CoO*_x_* samples (where nc-Nb is a normal-conducting spacer, realized by a thin Nb film below the critical thickness for superconductivity [8–9,56–58], serving to decouple the adjacent ferromagnetic layers) from aligned to crossed, i.e., non-collinear configuration of the two ferromagnetic films.

As the long-range triplet component is predicted to be generated from the singlet component mostly at the interface between the two ferromagnetic layers [15], the strength of the *T*_c_ suppression should be strongly dependent on the thickness of the F_1_ layer adjacent to the S-layer, as the singlet PWF decays with increasing distance from the S-layer. The main aim of the present work is to investigate the dependence of the triplet SSV effect on the F_1_ layer thickness. Such investigations are scarce so far [42,44,47].

The generation of spin-triplet Cooper pairs in S/F-heterostructures is expected to be the key to merge superconductivity and spintronics, because spin currents can be realized by supercurrents flowing through ferromagnetic materials, thus minimizing heating effects in spintronic devices [59]. Recently, a triplet SSV was employed to induce magnetism into a normal conducting metal [60]. Triplet SSVs are expected to perform logical functions like the normal conducting giant magnetoresistance (GMR) devices, however, with a much better energy efficiency [61].

## Sample preparation and characterization

**Figure 1:**
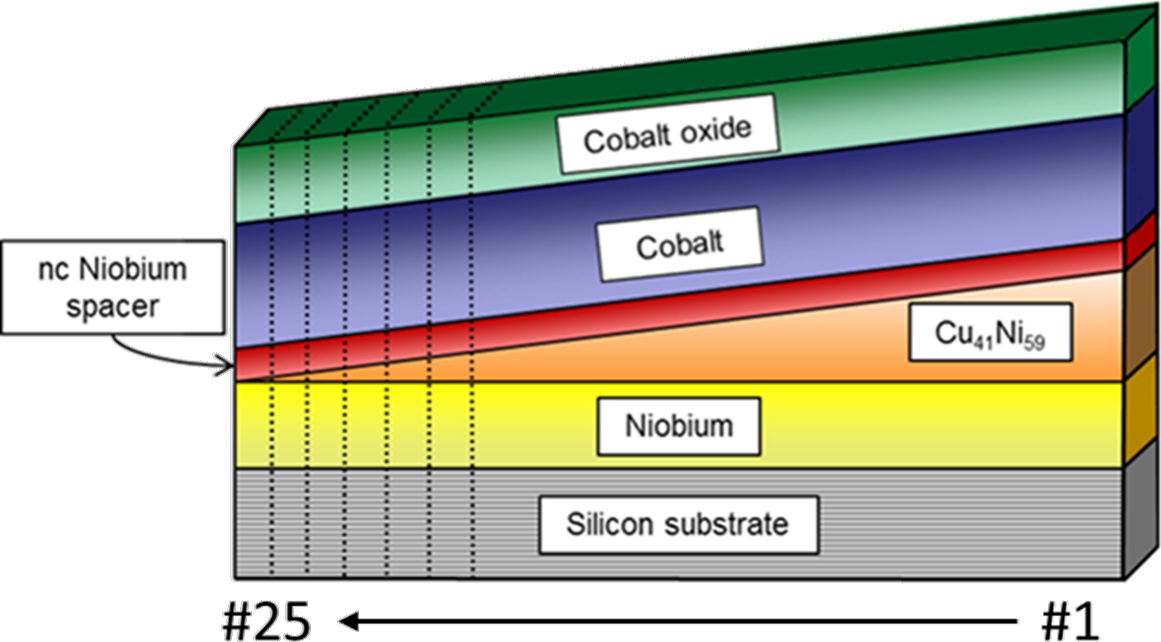
Sketch of the prepared Nb/Cu_41_Ni_59_/nc-Nb/Co/CoO*_x_* sample system SF_1_NF_2_-AF1. The dotted lines indicate the cutting procedures yielding the individual samples.

The sample series investigated in this work was prepared by magnetron sputtering at room temperature on a commercial silicon substrate with a {111} surface. While for the sputtering of the niobium layers, the target was moved over the substrate to obtain a smooth layer of nearly constant thickness, the Cu_41_Ni_59_ layer was deposited on the substrate positioned off-axis below the sputtering target to utilize the natural sputtering gradient of magnetron sputtering to obtain a wedge of varying thickness. Subsequently, the Co layer was deposited on the substrate without moving the target. Finally, the CoO*_x_* layer was sputtered from the same target as the metallic Co layer, however, oxygen gas was mixed into the chamber atmosphere to achieve reactive growth of cobalt oxide. For more details on the sputtering procedures see [8–9,41]. The heterostructure obtained was then cut perpendicular to the thickness gradient of the Cu_41_Ni_59_ wedge into 25 individual samples of roughly 2.5 × 8 mm^2^ size, yielding a set of Nb/Cu_41_Ni_59_/nc-Nb/Co/CoO*_x_* samples, with varying Cu_41_Ni_59_ layer thickness, which is virtually constant inside a single sample. By preparing all samples within the same run we ensured all sample parameters, which are influenced by the sample preparation (such as interface roughness, boundary resistance, etc.) to be the same for all prepared samples. Figure 1 shows a sketch of the prepared wedge, the dotted lines indicate how individual samples were cut to obtain the sample series.

**Figure 2:**
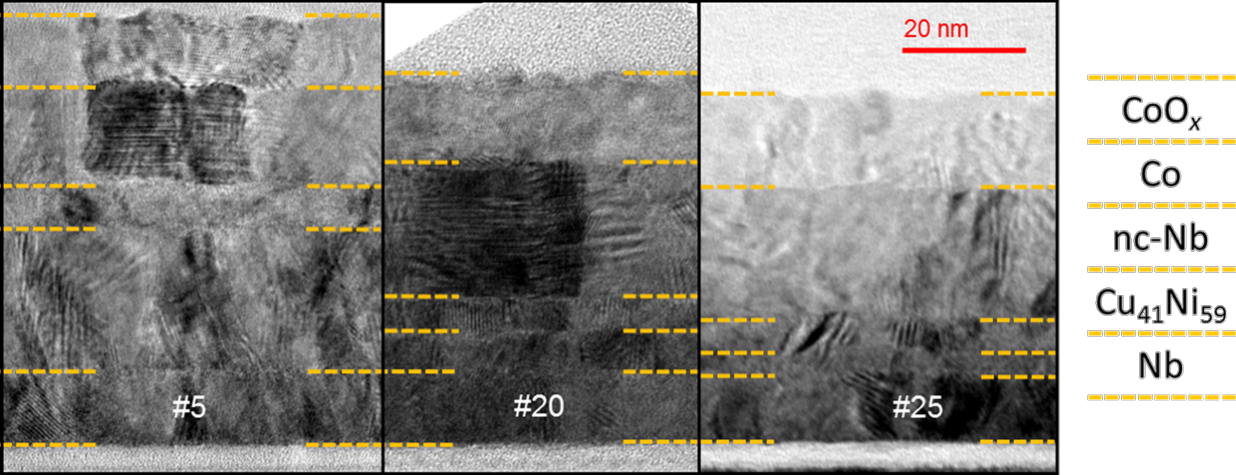
Cross-sectional TEM images of samples SF_1_NF_2_-AF1 #5, #20, and #25 from left to right. The dashed yellow lines indicate the interfaces between the layers. The obtained layer thicknesses are given in Table 1.

We investigated three samples (SF_1_NF_2_-AF1#5, #20, and #25) by cross-sectional transmission electron microscopy (TEM) to check correct deposition and to prove that the interfaces between the layers are clean and smooth. In Figure 2 examples of the obtained images for all three samples are shown. All layers are clearly distinguishable and have sharp and plain interfaces. Furthermore, the layer thicknesses determined by the TEM analysis are used as initial guesses to fit the Rutherford backscattering spectroscopy (RBS) spectra, from which the thicknesses of the layers of further samples are obtained. The thicknesses for the samples investigated by cross-sectional TEM are summarized in Table 1.

**Table 1:** Layer thicknesses of several samples of the investigated sample series SF_1_NF_2_-AF1 as obtained by a cross-sectional TEM investigation. Please note, that the thicknesses given are evaluated over a larger range than shown in Figure 2.

sample	layer thickness [nm]				
	Nb	Cu_41_Ni_59_	nc-Nb	Co	CoO*_x_*

#5	12	23	7	16	11.5
#20	12	6.5	6	22	14.5
#25	11	4	5.5	22	15.5

**Figure 3:**
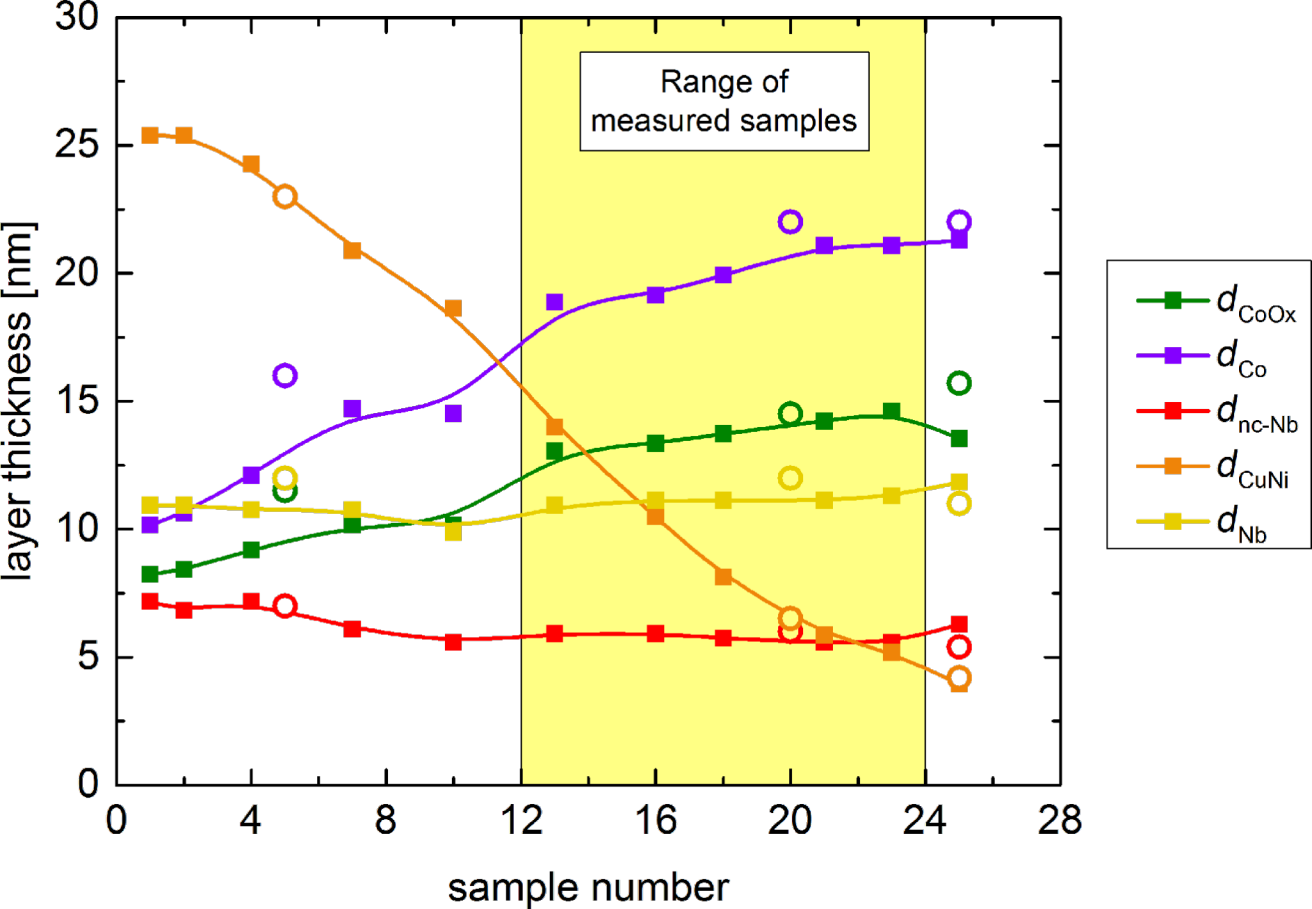
Thickness analysis of the sample series SF_1_NF_2_-AF1. While the solid squares show the data obtained by RBS analysis, the open circles are the values from TEM imaging. The solid lines are a guide to the eye.

About one half of the samples were investigated by RBS analysis. In Figure 3 the results for all constituent layers are shown. The layer thicknesses from the TEM analysis are shown as open symbols. Since the sensitivity of RBS for light elements is small, the adjacent Co and CoO*_x_* are virtually indistinguishable. Thus, the thickness values for Co and CoO*_x_* might be slightly different from those given in Figure 3. However, the thicknesses obtained by TEM investigations and linear interpolations between them were used as initial values for the splitting of the metallic and oxide cobalt signals, cross-checked with the RBS spectra for plausibility and slightly adapted to yield the best fit to the RBS spectra.

**Figure 4:**
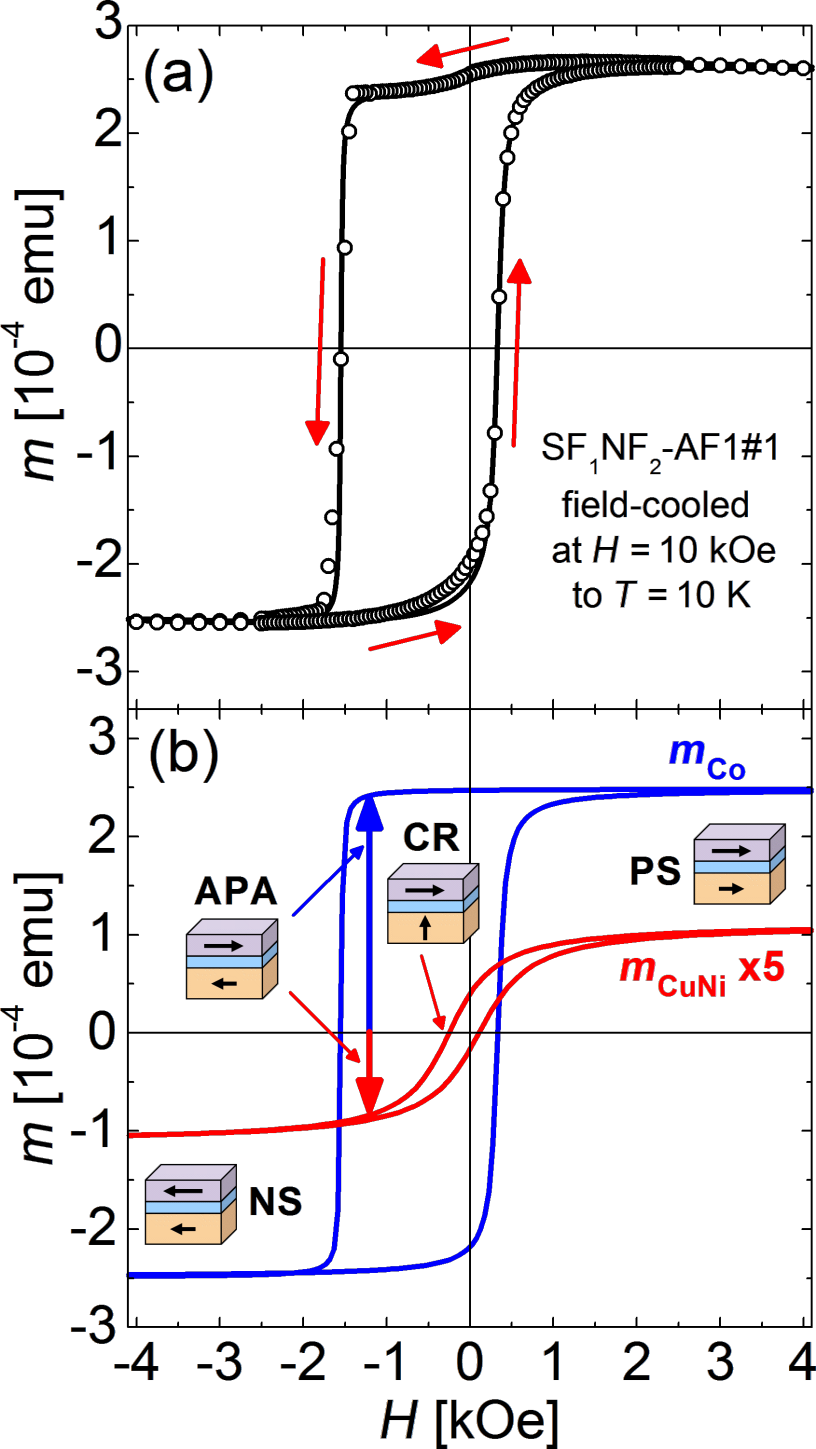
(a) Magnetic hysteresis loop of SF_1_NF_2_-AF1#1 recorded by a SQUID magnetometer. Here, *m* is the magnetic moment and *H* the magnetic field applied parallel to the film plane. The solid line shows a reconstruction of the hysteresis loop according to the simple version of the model of Geiler and co-workers [62]. The corresponding parameters are summarized in Table 2. The sample was cooled to 10 K in a field of 10 kOe applied parallel to the film plane. The red arrows indicate the sweep direction. (b) Separation of the obtained hysteresis loop into the constituent loops of the individual layers according to Equation 1. The copper–nickel signal is enlarged by a factor of 5 to improve visibility. The pictograms indicate the different magnetic configurations of the system (PS: positive (*H* > 0) saturated; NS: negative (*H* < 0) saturated; APA: antiparallel alignment of the magnetic moments of the F_1_ = Cu_41_Ni_59_ and F_2_ = Co layers; CR: crossed configuration of the magnetic moments of F_1_- and F_2_ layers). The diamagnetic contribution of the silicon substrate is subtracted (only in (b)). Here, cgs emu units are used. In SI units, it is 1 emu = 10^−3^ A·m^2^ and 1 Oe = 10^3^/(4π) A/m [63], so that (with μ_0_ = 4π·10^−7^ V·s/(A·m)) the magnetic flux density related to 10 kOe is *B* = μ_0_*H* = 1 T.

Furthermore, we conducted superconducting quantum interference device (SQUID) magnetometry investigations on several SF_1_NF_2_-AF1 samples (#1, #11, #13, and #16) after cooling down to 10 K in a magnetic field of 10 kOe, applied parallel to the film plane and perpendicular to the Cu_41_Ni_59_ gradient, to ensure correct exchange biasing of the Co layer and to determine the field ranges, at which the positive saturated (PS), negative saturated (NS), and crossed (CR) configuration, as well as an antiparallel alignment (APA) of the magnetizations are realized. Exemplary, Figure 4a shows the data for sample SF_1_NF_2_-AF1#1. The red arrows indicate the sweep direction of the applied magnetic field. The solid line represents a reconstruction of the hystersis loop based on the simple version of the model of Geiler and co-workers [62].

**Table 2:** Parameters used in modeling the hysteresis loop in Figure 4 according to the simple version of the model of Geiler and co-workers [62]. Here, negative and positive sweep direction refers to the semi-loops, where the applied field was decreased from positive saturation to negative saturation and vice versa, respectively. Moreover, a linear contribution with a slope of *s* = −2.98·10^−6^ emu/kOe and an offset of the magnetic moment scale of *m*_0_ = 4.4·10^−6^ emu are considered. For a critical discussion of the used parameters see the text.

layer	sweep direction	*m_s_* [10^−4^ emu]	*H*_c_ [kOe]	*H*_t_ [kOe]

Co	−	2.49	−1.551	0.015
Co	+	2.49	0.333	0.065
CuNi	−	0.22	−0.251	0.400
CuNi	+	0.22	0.108	0.450

To further visualize the two contributions to the solid line in Figure 4a, its decomposition into the two constituent ferromagnetic signals is shown in Figure 4b. For better visibility the Cu_41_Ni_59_ signal was enlarged by a factor of 5. The corresponding equation is given by

[1]
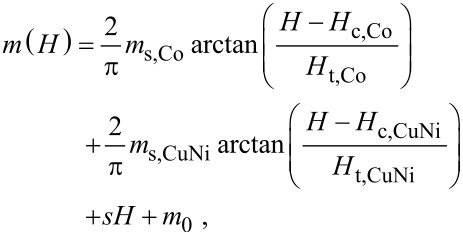


with *m*(*H*) being the magnetic moment, *m*_s_ the saturation magnetic moment, *H*_c_ the coercive field, and *H*_t_ a threshold field, determining the field (relative to the coercive field), at which half of the saturation magnetization is realized. The second subscript of the parameters denotes the corresponding material. Moreover, *m*_0_ is a small offset of the magnetic moment scale and *s* is the slope of the superposition of the diamagnetic and paramagnetic contribution from the substrate and the antiferromagnet [64], respectively. The parameters are summarized in Table 2. While the Cu_41_Ni_59_ loop is centered at almost zero field, the Co signal is clearly shifted to negative fields by the exchange bias interaction with the CoO*_x_*. A similar shift of the Co signal could be detected in all samples, however the weak Cu_41_Ni_59_ contribution is increasingly hard to evaluate, the thinner the Cu_41_Ni_59_ layer is.

We should remark, that the present reconstruction of the hysteresis loop is not unambiguous. Moreover, it shows small deviations from the data, especially for the positive sweep direction around *H* = 0. Possibly, these deviations can be reduced by the extended version of the model of Geiler and co-workers [62]. However, this requires the inclusion of three additional fit parameters per layer and sweep direction. This fact, in conjunction with the lack of clear structures in the hysteresis loop, yields mutual dependencies of the parameters and, thus, renders the extended model inapplicable. Moreover, even in the simple version of the model, the obtained parameters include an estimated error of about ±10% for *m_s_* and ±(10–20)% for the other parameters.

Investigations of the hysteresis loops of (Cu_41_Ni_59_/Si) × 4 samples show that the magnetically easy axis is directed perpendicular to the film plane [41]. This finding is supported by ferromagnetic resonance (FMR) measurements performed for these samples, as well as by our FMR measurements carried out for single Cu_41_Ni_59_ layers. The result is in agreement with the literature [53].

From the saturation magnetic moment, the magnetic moment per atom can be calculated according to the term *m*_at_/μ_B_ = [*m*_s_/(*V*_L_*N*_A_/*V*_mol_)](0.9274·10^−20^ emu)^−1^. Here, *V*_L_ is the volume of the respective layer, *V*_mol_ is the molar volume of the respective material, *N*_A_ the Avogadro constant and μ_B_ = 0.9274·10^−20^ emu [65] the Bohr magneton. For Cu_41_Ni_59_ and Co it is *V*_mol_ = 6.8 cm^3^ [9] and 6.62 cm^3^, respectively. The area of the sample SF_1_NF_2_-AF1#1 is 25 mm^2^. With *d*_CuNi_ ≈ 25 nm and *d*_Co_ ≈ 10 nm from Figure 3 and the respective saturation magnetic moments *m*_s_ from Table 2, we obtain *m*_at_ = 0.0429μ_B_ and *m*_at_ = 1.18 μ_B_ for the Cu_41_Ni_59_ and for the Co layer, respectively. The results for *m*_at_ are much smaller than the bulk material values of 1.7μ_B_[66] and 0.14μ_B_[67] for Co and Cu_41_Ni_59_, respectively, especially in the latter case.

For isolated Cu_41_Ni_59_ layers with *d*_F_ = 21 nm and *d*_F_ = 48 nm, investigated in our previous work [9], which were deposited directly on Si substrates by the same preparation methods used in the present work, *m*_at_ determined from *m*_s_ shows no reduction from the bulk material value. However, for Nb/Cu_41_Ni_59_ bilayers with *d*_Nb_ = 14.1, 13.0 and 14.2 nm and *d*_CuNi_ = 34.3, 26.5, and 18.8 nm, respectively, hysteresis curves measured by SQUID magnetometry at *T* = 10 K, where the sample is normal conducting, yield *m*_s_ values resulting in *m*_at_ = 0.074μ_B_, 0.080μ_B_, and 0.127μ_B_.

**Figure 5:**
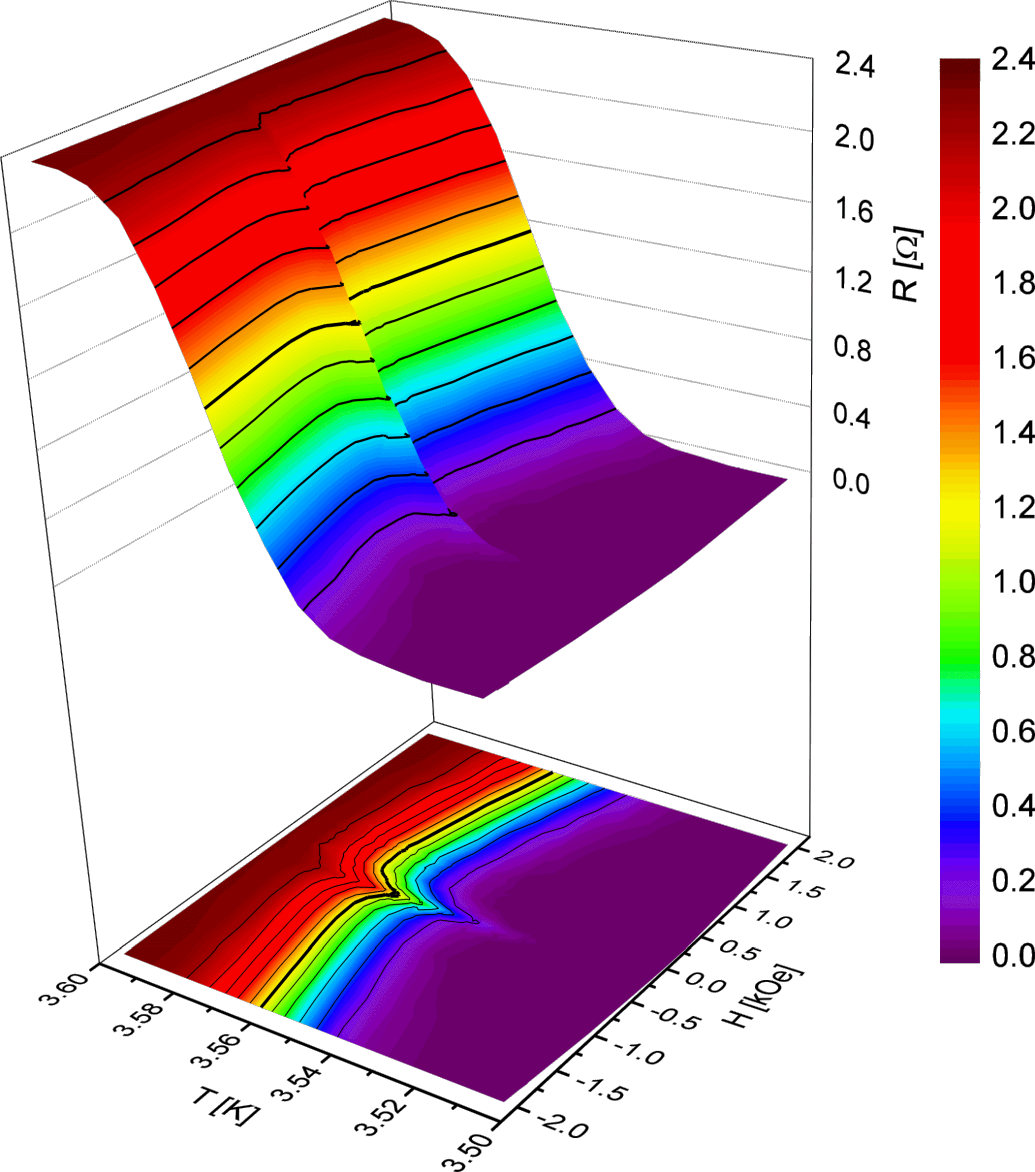
Superconducting transition as a function of the temperature, *T*, and the applied magnetic field, *H*, parallel to the film plane, obtained by four-probe measurements on sample SF_1_NF_2_-AF1#17 at a measuring current of 10 μA, both as 3D plot and planar projection. The applied magnetic field was varied from positive to negative values. The solid lines represent certain resistance levels, the bold line indicates roughly the level, where we evaluated the transition temperature, *T*_c_, at half of the normal-state resistance.

Investigations of the magnetic properties of thin films of Co (deposited on W) [68] and Cu_40_Ni_60_ (deposited as Cu_40_Ni_60_/Cu multilayers) [53], show that in both cases the magnetization is given by the bulk material value, so that the magnetic moment (for fixed area of the sample) increases linearly with the thickness of the magnetic layer (see Figure 1 and Equation 12 of [68] and Figure 5 and Equation 6 of [53]). However, a non-magnetic “dead” layer of thickness 0.18 nm and 2 × 1 nm at the interfaces to the W and Cu, respectively, is observed.

Such non-magnetic layers can in principle exist in the Nb/Cu_41_Ni_59_ bilayers (and also in the sample of the present work, where the Cu_41_Ni_59_ layer is sandwiched between two Nb layers). While the solubility of Nb in Cu is extremely small under ambient conditions, the Nb–Ni phase diagram shows a certain solubility of Nb in Ni and vice versa [69]. In this case the bilayer with the thinnest Cu_41_Ni_59_ layer would show the largest reduction of *m*_at_. However, the opposite is observed.

There may be several sources of experimental error in the absolute value of small magnetic moments present in SQUID measurements [70–71]. However, in the present study these effects should reduce *m*_s_ of both magnetic layers by the same factor, yielding the ratio between the saturation magnetizations being unaffected. With *m*_s_ = *m*_at_(*V*_L_/*V*_mol_)*N*_A_, where *V*_L_ = *A*·*d*_L_ with *A* and *d*_L_ the area of the sample and the thickness of the respective layer, using *m*_at_ = 1.7μ_B_ and 0.14μ_B_ for Co and Cu_41_Ni_59_, respectively, we obtain *m*_s,Co_/*m*_s,CuNi_ = 12.5(*d*_Co_/*d*_CuNi_). With *d*_Co_ ≈ 10 nm and *d*_CuNi_ ≈ 25 nm, we obtain a ratio of 5. Even if we take into account that the thickness of the Co layer, determined from TEM investigations on SF_1_NF_2_-AF1#5, possibly indicates a smaller slope of the thickness profile for small sample numbers than evaluated from RBS (see Figure 3) and assume *d*_Co_ ≈ 15 nm for SF_1_NF_2_-AF1#1, the ratio increases only to 7.5. This is much smaller than the ratio of 2.49/0.22 = 11.3 obtained from the saturation magnetizations given in Table 2.

Thus, while the reduced values of *m*_at_ observed for S/F bilayers discussed above, might be explained by a reduced value of *m*_s_ caused by experimental errors in the SQUID signal, the extraordinary small value of *m*_at_ of the Cu_41_Ni_59_ layer in the present work cannot be explained due to such effects only. Therefore, for this layer other mechanisms of reduction of *m*_s_ have to be discussed. It has to be considered, that during deposition of the samples in the present work oxygen gas was mixed into the atmosphere of the sputtering chamber during growth of CoO*_x_*. This can locally lead to the formation of antiferromagnetic NiO [65] in the already deposited Cu_41_Ni_59_ layer, yielding a reduction of the magnetic moment of the film. A degradation of Cu_41_Ni_59_ by oxygen has been discussed in our former work on AF-F/S/F and F/S/F-AF samples, where F = Cu_41_Ni_59_, S = Nb, and AF = CoO*_x_* [72]. The samples were produced by the same deposition method as in the present work, except that the substrate was heated up to 300 °C and 200 °C to deposit the AF bottom and top layer, respectively. In these investigations, for AF-F/S/F samples aging effects (possibly due to oxygen diffusion from the CoO*_x_*) are observed. The theoretical fitting of the experimental data (parameters given in Table I of [72]) yields an increase of the magnetic coherence length, ξ_F0_ (for a definition, see [7,9]) in the F-layers (corresponding to ξ_F1_ in the present work), which is probably indicating a decrease of the exchange energy, *E*_ex_. The reason for such a decrease of *E*_ex_ can be a weakening of the exchange interaction caused by the formation of NiO. An increase in ξ_F0_ is also observed in the F/S/F-AF sample series (see Table I of [72]). However, this series is investigated just after deposition. A possible source of oxygen in this case may be the CoO*_x_* layer deposition after deposition of the Cu_41_Ni_59_ layers. This is the same case in the present study, however, the CoO*_x_* layer is deposited at room temperature here.

## Results and Discussion

To investigate the triplet SSV effect, resistance–temperature (*R*(*T*)) measurements of the superconducting transition were recorded at different magnetic fields applied parallel to the film plane. The measurements were performed in an Oxford Instruments Heliox sorption-pumped ^3^He insert, applying the DC four-probe method for both polarities of the sensing current (10 μA) to eliminate thermoelectric voltages. The samples were cooled down to liquid helium temperature in a field of 30 kOe, applied in the same direction as in the preceding SQUID measurements. By sweeping the temperature at various fixed magnetic fields, the magnetic field dependence of the transition temperature, *T*_c_, was obtained for samples SF_1_NF_2_-AF1#12, #17, #19, #22, #24.

Figure 5 exemplarily shows a plot of the transition curves, obtained for sample SF_1_NF_2_-AF1#17. The solid lines represent certain resistance levels. The bold line is the resistance level, at which we evaluated the transition temperature, *T*_c_, at one half of the normal state resistance. There are no obvious peculiarities in the shape of the transition curves, even at the coercive field of Cu_41_Ni_59_. At this field the transition is shifted towards lower temperatures over the whole width of the transition.

**Figure 6:**
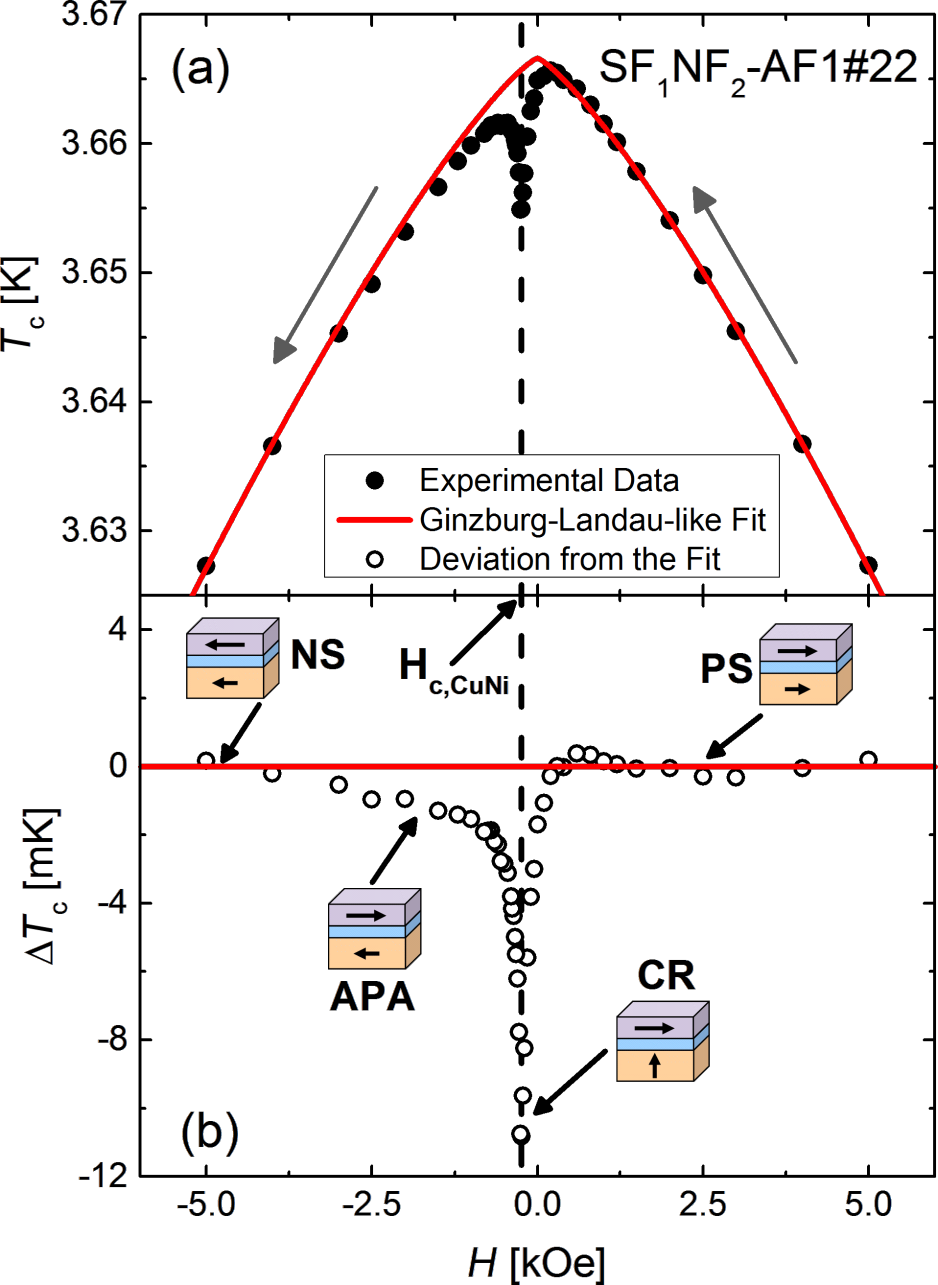
(a) Superconducting transition temperature *T*_c_ of SF_1_NF_2_-AF1#22 as a function of the applied magnetic field *H*. The direction of the variation of the applied field is given by arrows, the solid red line shows a fit to a Ginzburg–Landau-like behavior (for details see the text). (b) Deviation, Δ*T*_c_, calculated between the experimental data and the fit. The pictograms show the magnetic configurations of the system in accordance with the magnetic measurements in Figure 4. The maximal suppression at crossed configuration of the magnetic layers is evaluated as Δ*T*_c,max_. Here, *H*_c,CuNi_ is the coercive field of the Cu_41_Ni_59_ alloy layer.

Figure 6a shows the *T*_c_ data obtained for sample SF_1_NF_2_-AF1#22 as a function of the magnetic field. We observed a narrow and pronounced reduction of *T*_c_ at the coercive field of the Cu_41_Ni_59_ layer, which is already visible in the transition curves in Figure 5. We attribute this reduction to the theoretically predicted [15] reduction of the critical temperature by the generation of the long-range, odd-in-frequency, spin-projection one triplet component of superconductivity at non-collinear orientation of the magnetizations of the two ferromagnetic layers, F_1_ and F_2_, as discussed in detail in [41] and outlined in the Introduction section.

In Chapter III of [41], there is a detailed discussion excluding other possible origins of the observed phenomenon, e.g., stray fields generated by the multi-domain state of the Cu_41_Ni_59_ film at its coercive field. Moreover, recent investigations of a Nb/Cu_41_Ni_59_ bilayer show no evidence of a reduction of *T*_c_ neither at the parallel, nor at the perpendicular applied coercive field (see the comment at the end of Chapter IV.B.2 of [73]).

This behavior is representative for all investigated samples, however, the *T*_c_ reduction is varying in magnitude. To separate the triplet SSV effect from the reduction of *T*_c_ by the applied magnetic field, we approximate the temperature dependence of the parallel critical field by a Ginzburg–Landau (GL)-like behavior. According to [74], a superconducting thin film shows a temperature dependence of the upper critical field (parallel to the film plane) given by *H*_c_(*T*) = *H*_c_(0)(1 − *T*/*T*_c0_)^α^. Here, *T*_c0_ is the critical temperature, i.e., the superconducting transition temperature in the absence of currents and magnetic fields, and α is a parameter given by the effective dimensionality of the superconductor. We should remark, that strictly obeying the definition of *T*_c0_, it can not be defined for the heterostructures of the present work, because magnetic material is present. Here it is used to identify the transition temperature in zero external magnetic field. For a two-dimensional and a three-dimensional superconductor, α_2D_ = 1/2 and α_3D_ = 1, respectively. Identifying *T* with *T*_c_ and *H*_c_ with *H* (because we determine the transition temperature, *T*_c_ at fixed field *H*), we fitted a GL-like behavior given by

**Figure 7:**
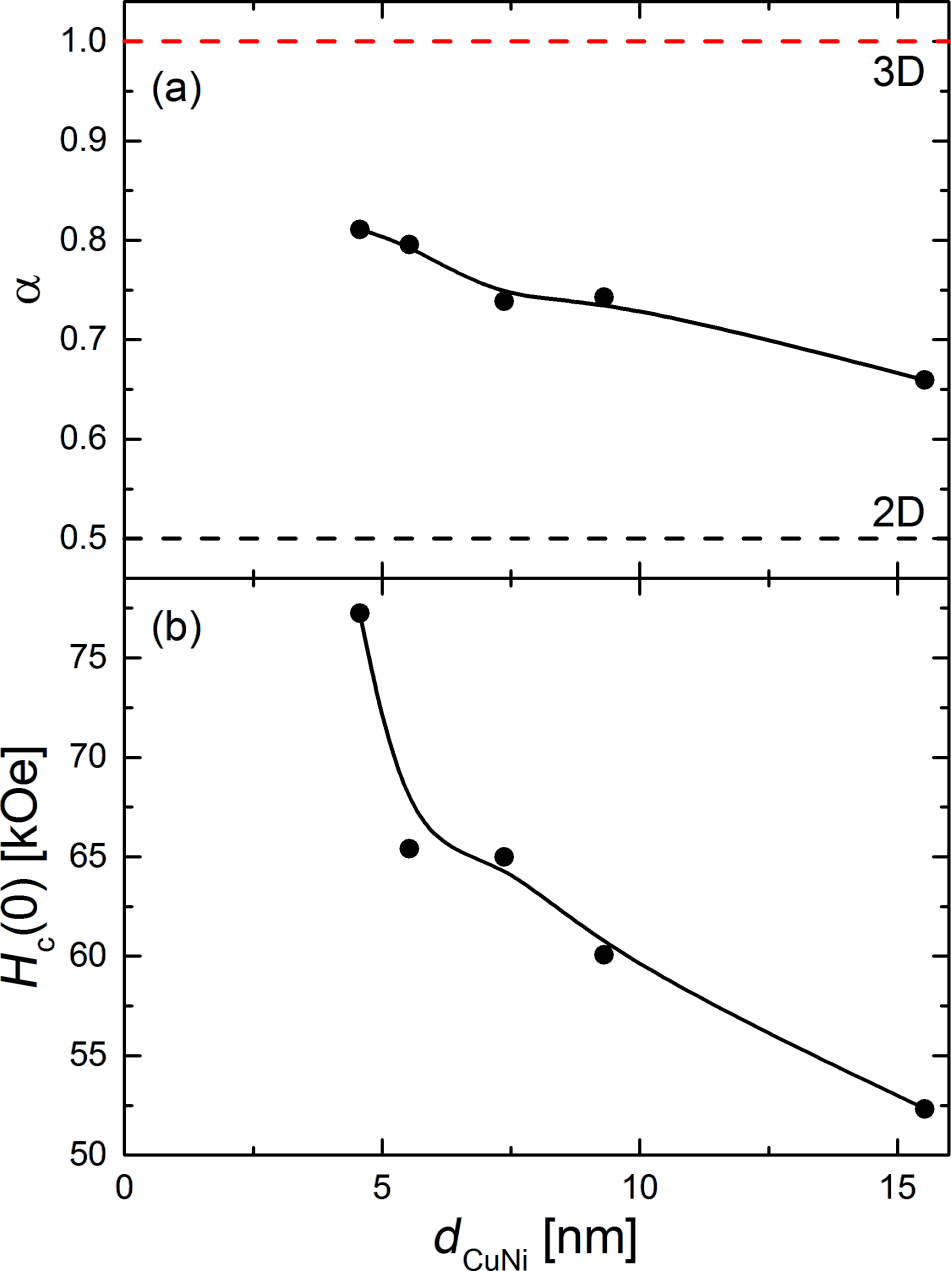
(a) Dimensionality parameter α and (b) the (fictive) upper critical field at zero temperature, *H*_c_(0), as obtained by fitting Equation 2 to the experimental data, as a function of the thickness of the Cu_41_Ni_59_ layer. The solid lines are guide to the eye, the dashed ones in (a) indicate the values of α for a two and a three-dimensional superconductor according to the GL-theory, respectively.

**Figure 8:**
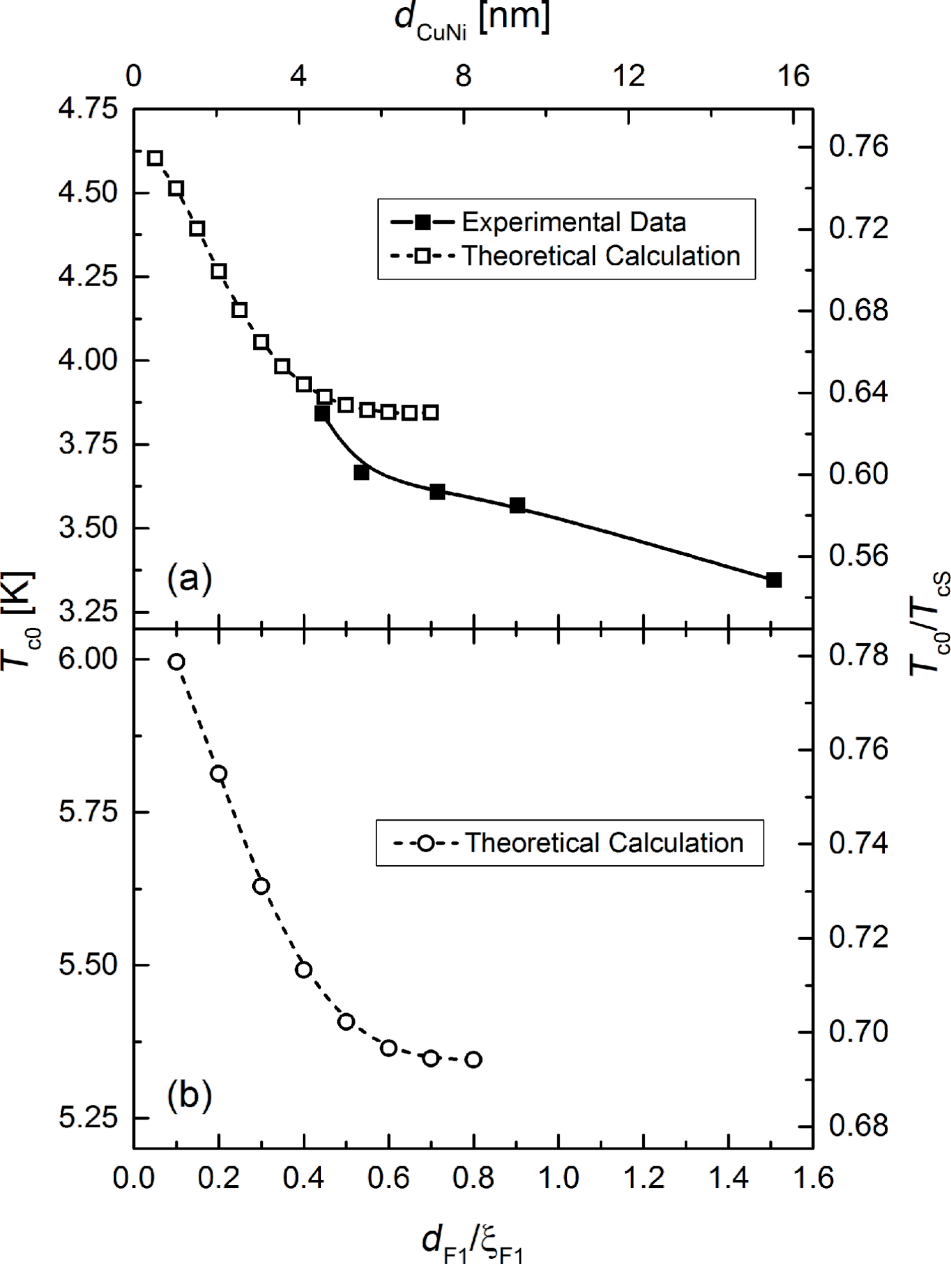
Superconducting transition temperature, *T*_c0_, in zero external magnetic field as a function of the F_1_ = Cu_41_Ni_59_ layer thickness *d*_F1_ = *d*_CuNi_. While the solid squares in (a) show experimental results, the open squares and circles in (a) and (b), respectively, show predictions, which can be derived from the theory of Fominov and co-workers [15]. The solid and dashed lines are guide to the eye. The theoretical data are plotted both in absolute and normalized (with respect to *T*_cS_) units. In (a), we assumed *T*_cS_ = 6.1 K, in (b) we used *T*_cS_ = 7.7 K, which is the critical temperature of a free standing Nb film of 12 nm thickness. Moreover, we used ξ_F1_ = 10.3 nm for the magnetic coherence length in the F_1_ layer (Cu_41_Ni_59_ layer) to plot the data as a function of the reduced thickness *d*_F1_/ξ_F1_. For a detailed discussion of the material and modeling parameters used for the theoretical calculations, see the Appendix.

[2]
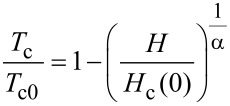


to the data points outside the area of reduced *T*_c_ and, thus, to data points corresponding to a parallel alignment of the magnetizations of the two ferromagnetic layers. Here, α and *H*_c_(0) and *T*_c0_ are fitting parameters, their dependencies on *d*_CuNi_ are given in Figure 7a and Figure 7b, and Figure 8a, respectively. All three parameters decrease as a function of *d*_CuNi_.

For *T*_c0_ this can be theoretically derived in this regime from the theory of Fominov et al. [15] (see Figure 8 for the calculated data, both in absolute units and normalized to *T*_cS_, the critical temperature of a freestanding Nb film of the same thickness as in the present sample series). Since *T*_c0_ decreases, this is also expected for *H*_c_(0). For the S-layer (for which usually 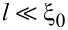 [73]), we obtain in the two-dimensional case 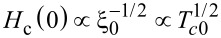 (see Equation 3 of [73]). Here, *l* is the electron mean free path and ξ_0_ the Bardeen–Cooper–Schrieffer (BCS) coherence length [74]. We do not expect that this is qualitatively different in the present samples.

However, the reason for the decrease of α is not so obvious. This decrease means that the effective dimensionality becomes more and more two-dimensional for an increasing thickness of the system, which is counter-intuitive. We can estimate for the S-layer, using typical parameters for our Nb films [9,73], that the GL coherence length is much larger than *d*_S_ in the range for which Equation 2 is applied. Thus, at least the S-layer is estimated to be in the two-dimensional limit.

**Figure 9:**
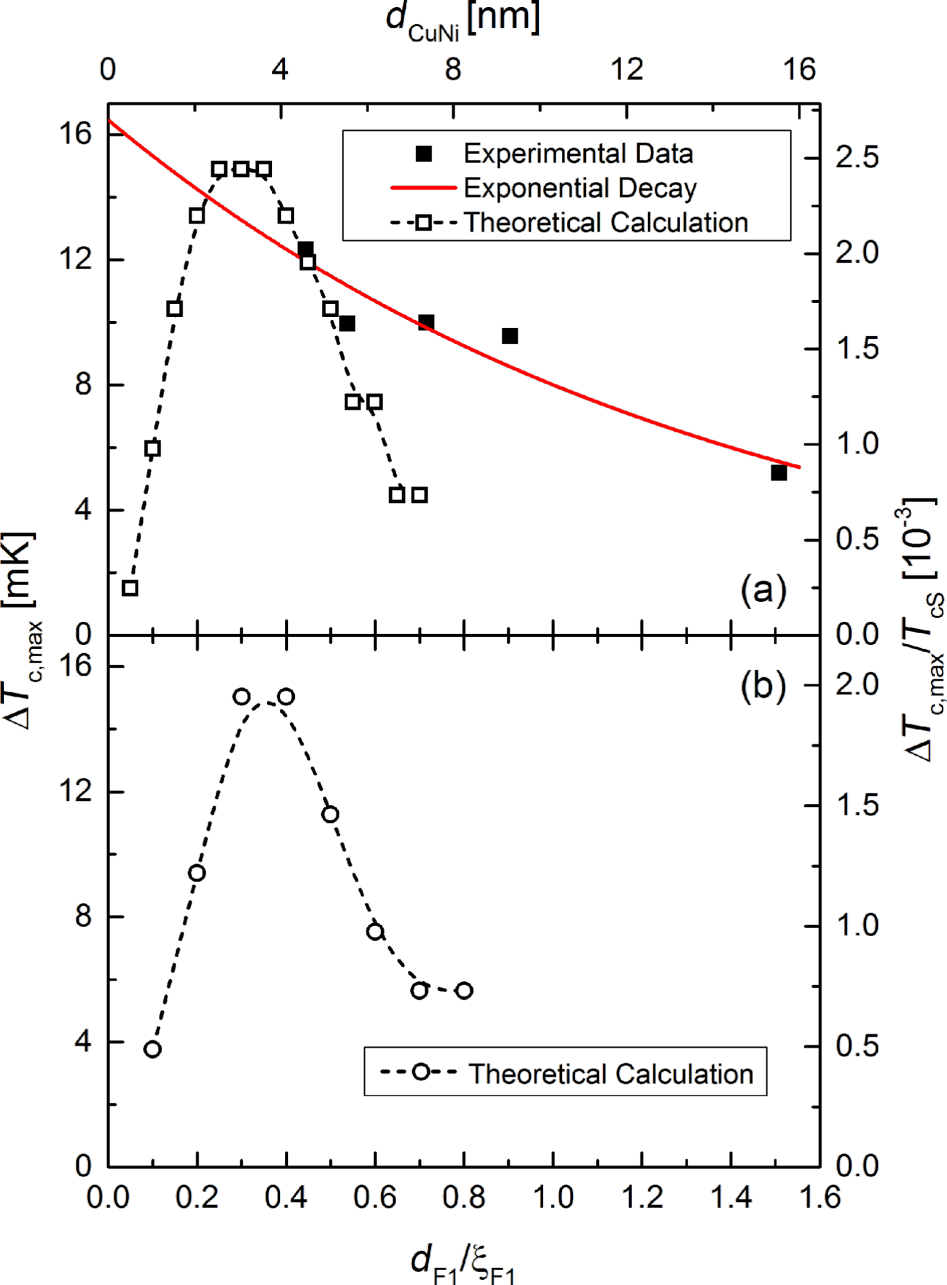
Maximum reduction of the transition temperature, Δ*T*_c,max_ by the triplet SSV effect at crossed configuration of the magnetic moments of the two ferromagnetic layers as a function of the thickness of the Cu_41_Ni_59_ layer. While the solid squares in (a) show experimental data, the open squares and circles in (a) and (b) show theoretical predictions as derived from the theory of Fominov and co-workers [15]. The theoretical data is plotted both in absolute and normalized (with respect to *T*_cS_) units. In (a), we assumed *T*_cS_ = 6.1 K, in (b) we used *T*_cS_ = 7.7 K, which is the critical temperature of a free standing Nb film of 12 nm thickness. The red line in (a) is a fit to an exponential decay according to Equation 3, using *l*_F1_ = 7.1 nm and Δ*T*_c,max_(0.6 nm) = 16.5 mK. Moreover, we used ξ_F1_ = 10.3 nm for the magnetic coherence length in the F_1_ layer (Cu_41_Ni_59_ layer) to plot the data as a function of the reduced thickness *d*_F1_/ξ_F1_. For a detailed discussion of the material and modeling parameters used for the theoretical calculations, see the Appendix.

The difference between the GL-like fit and the experimental data yields the net reduction of the transition temperature, Δ*T*_c_, without the pair breaking by the applied field. In the antiparallel relative alignment (APA) of the magnetization in the F-layers, the reduction of *T*_c_ is caused by a SSV effect, generated by the singlet and the spin-projection zero triplet components, while at non-collinear alignment, especially in the crossed configuration (CR), it is caused by the triplet SSV effect. The obtained values are given in Figure 6b as open circles. Since *T*_c_ in the APA state is smaller than predicted by the GL-like fit (corresponding to the positive or negative saturated (PS or NS) and, thus, parallel configuration), not a standard, but an inverse SSV effect [16] seems to be present. The maximum reduction in Figure 6b is evaluated as Δ*T*_c,max_ and plotted in Figure 9a as a function of *d*_CuNi_.

Fominov et al. [15] suggested the descriptive explanation that the triplet component is generated from the singlet component in the vicinity of the F_1_/F_2_ interface, where the Cooper pairs “experience” an inhomogeneous magnetization. The amplitude of the singlet component in the F_2_ layer depends on the thickness of the F_1_ layer, because the singlet component decays exponentially into the ferromagnetic material with a decay length of 2*l*_F1_, where *l*_F1_ is the electron mean free path in the material F_1_. Here, we assume that the singlet wave function of the FFLO state in the F_1_ layer is best described by the extension of the dirty-limit theory towards the clean case, as obtained for Nb/Cu_41_Ni_59_ bilayers [8–9]. In this case the decay length of the singlet state is given by 2*l*_F1_ (see the Appendix of [54], in [9] the factor of 2 was omitted). However, the singlet PWF in the present S/F_1_/N/F_2_/AF heterostructure not only decays in the F_1_ layer, but also in the N-layer, before reaching the F_2_ layer, i.e., it is reduced by an additional factor exp(−*d*_N_/ξ_N_). In our case, it is *d*_N_ = *d*_nc−Nb_ ≈ 6 nm (see Figure 3). As discussed in the Appendix, ξ_N_ = 20 nm is a suitable value. Thus, we assume that

[3]
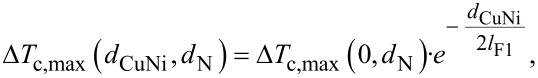


where 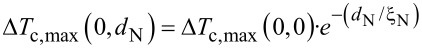. From the fit in Figure 9a, we obtain Δ*T*_c,max_(0,6 nm) = 16.5 mK, yielding Δ*T*_c,max_(0,0) = 22.2 mK.

The value of *l*_F1_ = 7.1 nm obtained from the fit is somewhat smaller than that in [9], where we obtained on average, that *l*_F1_/ξ_F1_ = 1.18 and ξ_F1_ = 10.3 nm, so that *l*_F1_ = 12.2 nm (in that paper *l*_F1_ and ξ_F1_ are denoted by *l*_F_ and ξ_F0_). This reduction of *l*_F1_ may be the result of the formation of NiO, which can serve as scattering centers, in the F_1_ layer (see section Sample Preparation and Characterization). In our former work [72] electron mean free path values of Cu_41_Ni_59_ layers down to 5.9 nm were observed for samples probably aged under the influence of oxygen (see Table I of [72]).

From the theory of Fominov et al. [15] follows a breakdown of the triplet SSV effect for small *d*_F1_, as shown in Figure 9. Apparently, for *d*_F1_ = 0 the system becomes a S/N/F_2_ structure (possibly due to proximity-induced superconductivity even a S/F_2_ bilayer) and, thus, the effect should be zero. The breakdown at small, but non-zero *d*_F1_ can be explained by the fact, that if *d*_F1_ becomes much smaller than the coherence length of the Cooper pairs in the F_1_ material, they should not be able to “experience” the exchange interaction of the F_1_ layer. For large *d*_F1_ the microscopic theory predicts a continuous decrease with increasing *d*_F1_. Our measurements seem to show the decreasing tail for *d*_F1_/ξ_F1_ beyond the maximum of Δ*T*_c,max_.

There is a qualitative agreement between theory and experiment. In Figure 8 the predicted decrease of *T*_c0_(*d*_F1_) is observed. However, in the experimentally investigated thickness range of *d*_F1_ the calculated decrease is much to small. Moreover, the temperature ranges of *T*_c0_ from experiment and theory only join, if *T*_cS_ = 6.1 K is assumed. This is considerably lower than expected for an isolated Nb layer of 12 nm from our former investigations [9]. Naturally, we can not be sure, that the deposition conditions of the Nb layer in the present experiment are identical, so that a somewhat different critical temperature is possible. Especially the reactive deposition of CoO*_x_* may decrease *T*_cS_ by decreasing the quality of the Nb film. A decreased critical temperature of the Nb film has also been assumed in our former work [72] to theoretically describe the experiments on samples, which were probably aged under the influence of oxygen (see Table I of [72], *d*_Nb_ is given in the figure captions, and Figure 5 of [9]).

In Figure 9, the decay of Δ*T*_c,max_(*d*_F1_) predicted by the theory is much stronger than observed experimentally. Both in Figure 9a and Figure 9b, the predicted absolute range of Δ*T*_c,max_ fit the experimental data. One possible reason for the observed deviations between the theoretical predictions and the experimental data is that the theory of Fominov et al. [15] is only valid for dirty ferromagnetic material (i.e., if 
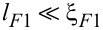
). However, neither the Cu_41_Ni_59_ layer nor the Co layer are in the dirty limit. While for Co this is obvious, we concluded in our former work [9], that Cu_41_Ni_59_ is between the dirty and clean limit (i.e., if 
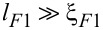
). Moreover, we also observed that the oscillations in *T*_c_(*d*_F_) are better described by an extension of the dirty-case theory towards the clean limit than by the dirty theory [8–9].

Moreover, the reduced atomic magnetic moment for Cu_41_Ni_59_ in our samples probably leads to a decrease of *E*_ex_ and, thus, an increase of ξ_F1_ beyond 10.3 nm found in investigations of S/F-bilayers [9]. In more recent investigations [72], values up to 17 nm are observed for Cu_41_Ni_59_ films, which were aged or exposed to an oxygen atmosphere after deposition of the Cu_41_Ni_59_ film. Using such a value to scale the experimental data, would yield the data point with the largest thickness of *d*_CuNi_ ≈ 15 nm to correspond to a reduced thickness of *d*_F1_/ξ_F1_ = 0.9, narrowing the gap between theory and experiment. However, since ξ_F1_ is connected to *E*_ex_, which influences further parameters of the calculation, for a final statement detailed theoretical calculations with increased values of ξ_F1_ and decreased *E*_ex_ need to be carried out.

Basically, the observed triplet SSV effect may be masked by the generation of a spin-projection one triplet component at domain walls (where the magnetization is also non-uniform) [49]. The occurrence of domain walls is expected just at the same applied field as the triplet SSV effect, i.e., at the coercive field [65]. This effect is not considered in the theory of Fominov and co-workers [15]. However, the multi-domain state should also be present in S/F-bilayers of Nb/Cu_41_Ni_59_ at the coercive field, at which no reduction of *T*_c_ was observed in our former work (see the discussion at the end of Chapter B.2 in [73]).

## Conclusion

In the present work we investigated the triplet superconducting spin-valve (SSV) effect in a S/F_1_/N/F_2_/AF heterostructure with S = Nb; F_1_ = Cu_41_Ni_59_; N = nc-Nb; F_2_ = Co; AF = CoO*_x_*. The experiments show a decay of the effect with increasing thickness of the F_1_ layer, *d*_F1_.

The microscopic theory predicts a breakdown of the effect for very thin F_1_ layers. Apparently, for *d*_F1_ = 0 the system becomes a S/N/F_2_ structure (possibly due to proximity-induced superconductivity even a S/F_2_ bilayer) and, thus, the effect should be zero. The breakdown at small, but non-zero *d*_F1_ can be explained by the fact, that if *d*_F1_ becomes much smaller than the coherence length of the Cooper pairs in the F_1_ material, they should not be able to “experience” the exchange interaction of the F_1_ layer. For large *d*_F1_ the microscopic theory predicts a continuous decrease with increasing *d*_F1_, as observed in the present work.

We modeled the experimental data both, with the microscopic theory, as well as an empirical model, which predicts an exponential decay with the decay length of 2*l*_F1_, where *l*_F1_ is the electron mean free path in the F_1_ material. This model is based on a simple picture, in which the reason for the decay of the triplet SSV effect with increasing *d*_F1_ is the exponential spatial decay of the singlet FFLO pairing wave function in the F_1_ layer. From this wave function the triplet component is generated mainly at the border of the F_2_ material.

The empirical model yields a quantitative agreement with the data with *l*_F1_ of the order of the electronic mean free path in Cu_41_Ni_59_. The decay of the effect with increasing *d*_F1_ is, however, only in qualitative agreement with the microscopic theory. For quantitative agreement between microscopic theory and experiment (if possible at all for clean, e.g., Co, or intermediate, e.g., Cu_41_Ni_59_, ferromagnets), a more detailed theoretical study of the influence of the coherence length and the exchange energy of the *F*_1_ material has to be performed.

## Appendix

### Material parameters of the theoretical calculations in Figure 8b and Figure 9b

The critical temperature of a free standing Nb film of 12 nm, as in our heterostructure, is 7.7 K according to Figure 5 of [9]. The coherence length ξ*_S_* = 6.68 nm [9] for a free standing Nb film of 14 nm thickness, which is close to *d*_S_ of the samples in the present work. Moreover, ξ_F1_ = 10.3 nm (average of the values ξ_F0_ given in [9]). For F_2_ = Co we take ξ_F2_ = 1.3 nm from [56] and *d*_F2_/ξ_F2_ = 15, i.e., *d*_F2_ = 19.5 nm, which is close to the midpoint of *d*_Co_ of the investigated samples (see Figure 3).

For the N spacer, the decay length of the PWF is given by [75] 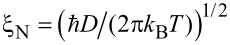, where *D* = (1/3)*l*_N_*v*_F_ is the diffusion constant. Here, *l*_N_ is the electron mean free path, and *v*_F_ is the Fermi velocity, 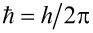 with *h* being Planck’s constant, and *k*_B_ the Boltzmann constant. As our N layer is nc-Nb, we take *v*_F_ = 2.768 × 10^5^ m/s, according to [76], and *l*_N_ = 2.1 nm, as determined for a 7.3 nm thick Nb film (which is close to the thickness, *d*_nc−Nb_ ≈ 6 nm, of the nc-Nb spacer in our heterostructure) in Appendix B.4 of [73]. For *T*, we insert 3.6 K, which is the midpoint temperature of the temperature range of the *T*_c0_ values in Figure 8a.

For the exchange splitting energy of the Cu_41_Ni_59_ layer we take *E*_ex,CuNi_ = 14.7 meV, as obtained in [77] for Cu_40_Ni_60_ films. As 1 eV = *k*_B_·11604 K and *T*_cS_ = 7.7 K, we get *h*_F1_ = *E*_Ex,CuNi_/(π*k*_B_*T*_cS_) = 7.0. For Co *E*_Ex,Co_ = 0.93–1.05 eV according to [78]. Taking *E*_Ex,Co_ = 1.0 eV we get *h*_F2_ = *E*_Ex,Co_/(π*k*_B_*T*_cS_) = 480.

Furthermore, we used the following proximity strength parameters as modeling parameters: γ_F1,S_ = 0.2, γ_N,F1_ = 1.0, γ_F2,N_ = 10, γ_B,F1,S_ =0.5, γ_B,N,F1_ = 0.2, γ_B,F2,N_ = 3 (see definitions in [79]).

### Material parameters of the theoretical calculations in Figure 8a and Figure 9a

Unless otherwise stated, the material parameters are the same as mentioned in the first section of the Appendix. The critical temperature of the free standing Nb film is assumed to be *T*_cS_ = 6.1 K, yielding *h*_F1_ = 8.8 and *h*_F2_ = 606. If we take into account an enhancement factor of the slope of the temperature dependence of the upper critical field in [9], we obtain the coherence length in the superconductor ξ*_S_* = 7.3 nm. According to Butler [80], this factor is 1.18 for Nb in the dirty limit.
